# The chorioallantoic membrane (CAM) assay for the study of human bone regeneration: a refinement animal model for tissue engineering

**DOI:** 10.1038/srep32168

**Published:** 2016-08-31

**Authors:** Inés Moreno-Jiménez, Gry Hulsart-Billstrom, Stuart A. Lanham, Agnieszka A. Janeczek, Nasia Kontouli, Janos M. Kanczler, Nicholas D. Evans, Richard OC Oreffo

**Affiliations:** 1 Bone and Joint Research Group, Centre for Human Development, Stem Cells and Regeneration, Human Development and Health, Institute of Developmental Sciences University of Southampton, Tremona Road, Southampton, SO16 6YD, UK; 2 Cancer Sciences Unit, Somers Cancer Research, University of Southampton, Tremona Road, Southampton, SO16 6YD, UK

## Abstract

Biomaterial development for tissue engineering applications is rapidly increasing but necessitates efficacy and safety testing prior to clinical application. Current *in vitro* and *in vivo* models hold a number of limitations, including expense, lack of correlation between animal models and human outcomes and the need to perform invasive procedures on animals; hence requiring new predictive screening methods. In the present study we tested the hypothesis that the chick embryo chorioallantoic membrane (CAM) can be used as a bioreactor to culture and study the regeneration of human living bone. We extracted bone cylinders from human femoral heads, simulated an injury using a drill-hole defect, and implanted the bone on CAM or *in vitro* control-culture. Micro-computed tomography (μCT) was used to quantify the magnitude and location of bone volume changes followed by histological analyses to assess bone repair. CAM blood vessels were observed to infiltrate the human bone cylinder and maintain human cell viability. Histological evaluation revealed extensive extracellular matrix deposition in proximity to endochondral condensations (Sox9+) on the CAM-implanted bone cylinders, correlating with a significant increase in bone volume by μCT analysis (p < 0.01). This human-avian system offers a simple refinement model for animal research and a step towards a humanized *in vivo* model for tissue engineering.

Bone fracture is a major socio-economic burden that is set to rise as a consequence of current additional health issues including smoking, diabetes as well as an increasing ageing population^1^,^2^. Currently, approximately 10% of fractures fail to heal properly resulting in delayed union or non-union^3^,^4^,^5^. Poor vascularisation is frequently a contributing factor in impaired healing^6^ and thus a central challenge in bone tissue engineering is the design and development of biomaterials which can promote vascularization and aid bone repair.

To achieve this, a number of strategies have focussed on functionalised biomaterial scaffolds. These may harness growth factors, cells and small peptides, providing an osteogenic environment together with appropriate mechanical support to promote and guide tissue growth^7^. As a consequence the number of biomaterial combinations has increased significantly in the field of bone tissue engineering, evidenced by the increase in publications from 60 to over 600 per year in the last 15 years. However, the efficacy and safety of these biomaterials still requires extensive assessment prior to Food and Drug Administration (FDA) approval for clinical applications.

Biomaterial preclinical testing involves an initial evaluation of the cytotoxicity, function and proliferative effects of the biomaterial on cells, followed by further studies using animal models^8^. *In vitro* systems are often limited as predictors of clinical function as such models cannot fully reproduce the complexity of biological systems (blood supply, immune function, inflammatory and hormonal response), nor the complex interactions of different cell types. Therefore, the application of animal models to determine the efficacy of a material and the generation of safety/toxicity data prior to clinical evaluation is still required. However, discrepancies in experimental design (animal age, physiology, injury location and size, and bone composition) have resulted in the lack of reproducible standard animal models for bone tissue engineering. Furthermore, the inconsistent response of animals to drugs and devices in comparison to humans remains a concern^9^,^10^. For instance, bisphosphonates (inhibitors of bone resorption) increase bone mineral density in post-menopausal women, while the effects in animal models were reported to be bone site-dependent^11^. Such limitations, combined with a need to meet the ethical obligations to reduce, refine and replace (3Rs) animal usage in animal research^12^, underline the importance of the development of new models that avoid the need for animal experimentation and recapitulate robustly species-specific effects.

One potential approach to refinement animal experimentation is the use of the chorioallantoic membrane (CAM) assay, which involves the implantation of a material or compound on the extraembryonic membrane of the developing chick egg. Critically the CAM is not innervated and thus no pain is experienced by the chick. The chick embryo develops over 21 days and from day 4 the CAM forms, growing exponentially until day 14 (6 cm^2^ up to 65 cm^2^) to serve as a respiratory organ with a rapidly developing vascular system^13^. The CAM assay is commonly used to perform angiogenic (or anti-angiogenic)^14^,^15^,^16^ studies as well as multi-species graft transplantation^17^,^18^,^19^, given the partial immune-deficiency of the CAM. Studies indicate the production of immune cells (lymphocytes T and B) commences at day 11, however, the immune cells do not become fully mature until the embryo hatches (day 21)^18^,^20^,^21^.

The CAM assay has been used in tissue engineering for over 40 years as a non-innervated vascular bed^21^ and, thereby, as an intermediate step in between *in vitro* and the *in vivo* models. Unlike other *in vivo* models, such as the murine subcutaneous implant, the CAM assay is minimally invasive to the chick embryo and hence is a refinement model for animal research^12^. A number of studies have evaluated material constructs on the CAM not only to examine the angiogenic response but also as an indicator of the material-tissue biocompatibility. A plethora of materials have been examined, including collagen, silk and alginate^22^,^23^,^24^,^25^,^26^, as well as polymer-based constructs such as polycaprolactone (PCL), polylactic acid (PLA) and poly(lactic-co-glycolic acid) (PLGA) with and without factors or cells^27^,^28^,^29^. Despite these studies, there remain few examples that have attempted to measure the tissue repair process *ex vivo* on the CAM. In 2004, Yang *et al*. demonstrated the potential of a BMP-2-loaded PLA scaffold to fill a chick femur segmental defect using the CAM assay^30^. The authors showed new cartilage and matrix deposition, proving the capability of the femur to grow *ex vivo* using the CAM^30^. Similar results, achieved from experiments conducted on human tissue, would validate the CAM assay as a clinically relevant platform to study bone regeneration.

In the present study, we have tested the hypothesis that the CAM model can support the incubation of living human bone and provide a bioreactor for the study and understanding of bone regeneration *in vivo*. We used femoral head bone samples from human subjects undergoing orthopaedic surgery and developed a bone repair model by introduction of a small drill defect in the bone cylinders. The implantation of bone cylinders on the innervated CAM provides a system to mimic the vascular contribution to human bone healing – something not achievable with current *in vitro* models, and hence an alternative model for animal research. Moreover, the CAM offers a step towards the development of a humanised, predictive preclinical model. A pictorial overview of the experimental approach adopted is shown in Fig. 1.

## Results

### The CAM blood vessels integrate and infiltrate throughout the implanted human bone cylinders

We first examined whether freshly isolated human bone, implanted on the CAM of the chick egg as described in the methods section, integrated within the tissue of the CAM. Bone cylinders were incubated on the CAM or at the air-liquid interface of growth medium (*in vitro;* Fig. 2A) for 7 days. The chick embryo survival rate was 72.5% ± 20.61 SD at the end of the implantation period (n = 8–10, 4 independent experiments, Supplementary Table S1). The gross appearance of bone cylinders was not altered following 7 days culture *in vitro* (Fig. 2B,C) while the CAM-implanted bone cylinders were extensively surrounded by CAM tissue after 7 days implantation (Fig. 2I,J) with evidence of chick blood circulation around the graft site at the time of harvest (Supplementary Video S1).

Human and avian erythrocytes displayed different histological features, which facilitated the distinction of blood vessel origin: avian cells displayed a fusiform shape and nuclei (Supplementary Fig. S1B,D) compared to enucleated and biconcave mammalian red blood cells (Supplementary Fig. S1A,C). As expected, only human blood vessels containing enucleated erythrocytes were present in bone cylinders cultivated *in vitro* (see dashed arrow Fig. 2F). CAM blood vessels were clearly distinguishable macroscopically during harvest (Fig. 2H–J), as well as microscopically during histological evaluation. The CAM capillaries were apparent at the periphery of the bone cylinder, throughout the trabeculae and in close proximity to the central defect region of the human tissue (see arrows Fig. 2K–N). Thus, extensive CAM integration with the bone cylinders was evident by the infiltration of the surrogate blood supply into the human tissue.

### Human tissue remains viable following culture on the chick CAM

To determine whether human cells remained viable following CAM implantation or *in vitro* culture, primary cultures of human cells were derived from bone cylinders incubated for 7 days on the CAM, *in vitro* or maintained at 4 °C (negative control). To achieve this, chick embryos that constitutively expressed green fluorescent protein (GFP) were used in order to distinguish between host (GFP-CAM) and human cell outgrowths. Initial cell outgrowth from explanted bone samples was evident both in the *in vitro* and CAM-implanted group after 9 days and 4 days, respectively; however, no cells were detected from the bone cylinders maintained at 4 °C. No green fluorescence was observed in cells derived from the bone cylinders cultured *in vitro* (Fig. 3A–D).

Both GFP+ and GFP− cells were evident in the CAM-implanted group, indicating differences in the origin of the cells: avian and human (Fig. 3E,F). There were morphological differences in the appearance of GFP+ compared to GFP− cells. The GFP− cells displayed a fibroblastic, spindle-like morphology both in CAM-implanted and *in vitro* culture (Fig. 3A,E), as well as in the bone control explanted at day 0 (Fig. 3M,N). In contrast, chick GFP+ cells displayed variable morphology and size (Fig. 3E,F,I,J). The number of GFP+ cells in the CAM-implanted group was observed to decrease towards the end of the incubation period with a majority of GFP− cells growing in the well. Flow cytometry analysis indicated the presence of a mixture of GFP+ cells and GFP− cells in the CAM-implanted samples after 20 days in culture, with 29.75% GFP+ and 69.35% HLA+ (Fig. 3G,H). In all cases, GFP− cells stained positively for HLA, confirming their human origin. Bone control and *in vitro* culture groups showed over 99.7% of GFP− and over 99% HLA+ cells (Fig. 3A–D,M–P). In summary, these results indicate that human cells from the bone cylinder remained viable after 7 days incubation on the CAM, supporting the notion that CAM incubation promotes the continued survival of explanted cells.

### CAM-implantation induces the deposition of extracellular matrix (ECM) and endochondral condensations within human bone tissue

Histological examination of the CAM-implanted bone cylinders demonstrated close interaction between human and avian tissues (Fig. 4A–D). CAM infiltration was evident in the marrow space of the bone, as evidenced by the deposition of ECM (Fig. 4A) and the presence of avian blood vessels (arrows Fig. 4B). Alcian Blue and Sirius Red staining (Fig. 4A) showed deposition of matrix rich in proteoglycans, within which trabecular bone was encapsulated. To interrogate further the origin of the cells laying down the new extracellular matrix in the bone tissue, GFP immunostaining was used to identify the avian cells (Fig. 4C). GFP+ cells were ubiquitously present in the CAM and were observed in the bone marrow space, further validating the invasion of the avian membrane into the human tissue. Furthermore, the GFP+ cells were observed to be co-localized with areas of ECM deposition in the bone marrow (Fig. 4A,C). *In vitro* cultured bone cylinders did not show any evidence of ECM deposition or GFP staining (Supplementary Fig. S2).

In addition to the close association of human and CAM tissue, cell condensations were found within CAM tissue in close proximity to the human tissue (Fig. 5A). These condensations were composed of layers of tightly packed cells located between the avian and human tissue (Fig. 5B–E). Antibodies specific to GFP demonstrated the avian origin of the cell nodule (Fig. 5C). In addition, the GFP+ cells located within the nodule specifically stained for Sox9, a transcription factor expressed by chondroprogenitors (Fig. 5E). Another marker of cartilage matrix, collagen type II, was found in the surrounding ECM of the cell condensation nodule (Fig. 5D. These results indicate the invasion of avian cells (GFP+) into the bone cylinder, which co-localised with new matrix deposition and cell condensations expressing chondrogenic mesenchymal condensations.

### Mineral dense tissue is deposited in CAM-implanted human bone cylinders

Next, to determine if the incubation of human bone on the CAM facilitated bone mineral deposition, we measured changes in density using μCT. Bone cylinders were scanned pre and post incubation under identical scanning conditions. As an internal standard control, bone cylinders were kept at 4 °C and scanned at the same time points as the experimental groups. Bone cylinders implanted on the CAM for 7 days exhibited a significant increase in bone volume (6.9% ± 1.6 SD; p < 0.01) compared to the *in vitro* group (−1.5% ± 1.7 SD) and control (0.008% ± 1.3 SD) (Fig. 6C). Data representative of four independent experiments, using both osteoarthritic (OA) and osteoporotic (OP) femoral heads (Supplementary Fig. S3).

To determine the nature of the tissue driving the change in the bone volume measurements, the μCT cross-sections were further analysed applying a multi-level segmentation technique (see methods for more detail) instead of a standard binarisation (*i.e.* global thresholding) (Fig. 6). Multi-level Otsu thresholding allowed the differentiation between low (light grey) and high-density tissue (dark grey) and background (black; Fig. 6E). The relative bone volume change of each segment (low *versus* high density bone) was quantified in the CAM-implanted and *in vitro-*cultured bone cylinders as shown in Fig. 6F. A significant increase in the low density tissue (12.72% ± 5.4 SD; p < 0.001) was observed, with negligible changes in the higher density tissue (−0.26% ± 4.2 SD). In contrast to the effect shown in the CAM group, the *in vitro* treated bone cylinders displayed a decrease in the low density tissue (−3.02% ± 2.3 SD) and a negligible variation in high dense tissue (0.345% ± 1.9 SD). When comparing the global and multi-level methods of analysis, there was almost a two-fold difference between low density (12.72% ± 5.4 SD, Fig. 6F) and global (6.9% ± 1.6 SD, Fig. 6C) changes in bone volume in the CAM-implanted cylinders. Both methods (global and multi-level), reflected similar results in the *in vitro* cultured bone cylinders: negligible or negative change (Fig. 6C,F). These results indicate that low density material deposition drove the bone volume changes in the CAM-implanted bone cylinders.

Three dimensional registration software was used to visualise structural changes from μCT analysis of bone cylinders pre and post incubation. Co-registration paired images delineated matched (grey) and mismatched tissue (white and black) of the pre and post scans, respectively. On overlay, correct alignment of the bone cylinder was shown in most of the structure (grey colour in Fig. 7C,F,I). An example of divergence, resulting from actual physical change of the bone cylinder in the pre with respect to the post scan, is shown in the area highlighted by the green circle (Fig. 7G–I). Thus, the software was able to detect movement due to the location change of the trabecular bone spicule (Fig. 7I). One other structure was identified in the co-registered image as divergence in the bone cylinder defect area (see area highlighted in red in Fig. 7C,F,I). The overlay software displayed the structure in black colour (Fig. 7I), absent on the pre scan (Fig. 7G) and present in the post scan (Fig. 7H). Three independent observers assessed the overlay scan in 3D rendering to give an objective view, indicating the ability to visualise changes in the bone cylinder following incubation and, in particular, the deposition of new mineral-dense structures.

## Discussion

The current study has demonstrated the potential of the CAM as an *ex vivo* bioreactor for the culture of human bone tissue providing a surrogate blood supply. Integration of the human bone tissue with the CAM was evident by macroscopic and microscopic evaluation, with extensive penetration of avian capillaries into the human bone tissue. The human tissue was observed to remain viable after 7 days cultivation on the CAM membrane, similar to human bone cultured *in vitro*. CAM-culture of human bone cylinders elicited new extracellular matrix deposition and formation of endochondral cell condensations in the human tissue. The histological changes were further validated using μCT, which showed a significant and consistent increase in the bone volume (p < 0.01) after 7 days of incubation on the CAM.

To our knowledge, this is the first report of the culture of viable human bone tissue on the CAM in combination with a sophisticated μCT analysis to examine tissue regeneration. A previous study has implanted human bone on the CAM and examined the angiogenic response of the bone chips after each stage of the allograft banking procedure^31^. The authors did not report invasion of avian blood vessels in the graft and showed minimal CAM-integration of isolated bone debris^31^. In comparison, here we demonstrated infiltration of CAM capillaries through the human tissue with extensive CAM-integration of the entire bone graft. These striking differences could be explained by i) the length of the CAM-implantation period (2 days by Holzmann *et al*.^31^ compared to 7 days in the present report) and, ii) dissimilarities in the methodology (*i.e.* bone extraction, CAM assay).

The potential of this human-avian system to study bone regeneration was evidenced by the viability of human cells following GFP-CAM implantation. Human (GFP−/HLA+) cell outgrowths displayed a spindle-like and elongated shape morphology, typical of skeletal cells^32^. *In vitro* treated bone cylinders demonstrated a similar cell morphology after explant culture. Interestingly, cell outgrowth from the bone tissue was slower (9 days) compared to cell outgrowth from CAM-implanted cylinders (4 days). We hypothesise that the CAM growth impacted on the autologous human tissue, resulting in i) enhanced cell survival as a consequence of increased nutrient supply and ii) enhanced cell outgrowth as a consequence of matrix remodelling, which was not seen *in vitro*. Thus, the cell response from the human bone grafts was enhanced following CAM-implantation in comparison to *in vitro* culture.

Despite the use of xenograft material on CAM, embryo viability was reduced by only 27.5% compared to approximately 30% in other CAM studies^33^,^34^, supporting the concept that the CAM offers a biocompatible model for explant culture^22^,^33^. Moreover, a number of studies have shown the chick embryo is able to elicit a primitive immune response^20^,^21^,^35^, further highlighting the potential of the CAM assay as a tool to evaluate graft biocompatibility. Valdes *et al*. reported the presence of heterophils, leukocytes and giant cells as well as compact fibre deposition following 1, 7 and 11 days implantation of cotton threads, which the authors described as a chronic fibrotic response^22^. In contrast, our study showed loose matrix deposition, rich in proteoglycan and collagenous content, strongly associated with the presence of the avian cells (GFP+). The retention of embryo viability and the absence of a sustained fibrotic response support the conclusion that the human bone tissue can integrate with and is nourished by the CAM membrane.

Interestingly, we found evidence of cell condensations strongly expressing Sox9, an essential transcription factor expressed by chondroprogenitors^36^, in the CAM tissue in proximity to the human bone cylinder. Moreover, these cells also co-stained with GFP, confirming their avian origin. Similar cell condensations were shown by Hancox, who implanted chick embryo calvaria fragments on CAM for 10 days^37^. In accordance with our findings, cell condensations (named by Hancox ‘ectodermal pearls’) were located in proximity to the bone tissue^37^. These findings are not surprising given the clinical gold standard biomaterial is bone autograft^38^, which provides a unique osteogenic microenvironment as a consequence of the plethora of growth factors within the bone matrix (*i.e.* BMP, PDGF, FGF) as well as the presence of viable osteogenic cells^38^. Hence, it is likely that the implantation of such an osteoinductive material (*i.e.* living human bone cylinder) on the CAM can result in ectopic bone formation; indeed, it is well known that BMP proteins have significant structural similarity and function across species^39^.

The observation of new bone or cartilage formation occurring on the CAM is supported by our μCT data, which showed significant increases in the bone volume (p < 0.01) over the 7 day incubation period. We hypothesise that the changes in bone volume were driven by: i) the deposition of new low density matrix, possibly through CAM-derived ectopic bone formation, as evidenced by the histology; and ii) mineralisation of the pre-existing trabecular bone. Segmentation of the grayscale datasets into three levels (multi-level thresholding)^40^,^41^ showed a greater increase in the low density tissue (p < 0.001) compared to global thresholding (p < 0.01). It is worth considering that, even if bone volume was calculated as a relative change, multi-level thresholding was different for each bone cylinder, whereas global thresholding was applied equally to all bone cylinders. Critically, multi-level analysis allowed adaption to the subtle differences amongst the bone cylinders (structure, mineral content, composition: cortical *versus* trabecular) and thus, enhanced sensitivity when measuring specific regions of the grayscale histogram (*i.e.* low *versus* high density). Together, this data indicates that Multi-level Otsu thresholding allowed quantification of subtle changes in poorly mineralised tissue and that this less dense mineral tissue may be the main contributor to the increased bone volume measured using standard global thresholding.

In contrast to the increase in bone volume observed on the CAM-implanted bone cylinders, *in vitro* cultured samples showed a reduction in bone volume. This was despite the observation of a similar level of cell outgrowth and cell viability after 7 days incubation. In contrast, Kanczler *et al*. reported substantial mineralisation of early stage chick femurs following 10 days organotypic culture^42^. This mineralisation process occurred in the absence of angiogenesis due to it being a healthy and rapidly growing embryo organ. The present study evaluated bone from elderly patients with either osteoarthritis or osteoporosis, which is likely to grow significantly more slowly than embryonic bone and in all probability display an altered metabolic (catabolic) profile. These results support the conclusion that the CAM assay provides a unique vascular component in which bone tissue formation can be evaluated *in vivo*.

Given that most *in vivo* studies start to observe mineral deposition 14 days post-fracture^43^,^44^, extended incubation periods would be required to shed more light on this CAM-human model. The limitation of the short incubation period was addressed in the present study by implementing high resolution computed tomography, which allowed quantifying the subtle changes resulting of the early stages in bone regeneration. In addition, previous studies have shown that it is possible to observe bone formation within a 7 day timeframe when implanted on CAM^30^. It is important to note that the fetal chick skeleton becomes mineralised in the time period of 10 days^45^,^42^, thus, it is unsurprising that in our studies we observe mineralisation during the 7 day period of experimental measurement. The rapid and aggressive growth of the extraembryonic membrane has been previously demonstrated by Steffens *et al*., who compared the vasoproliferative response of the CAM model with the subcutaneous mouse implant^46^. Interestingly, the authors reported micro-vessel density was higher in the 7 day CAM assay (50.4 ± 17.3 SD), compared to the 21 days implantation in the murine model (43.5 ± 16.3 SD)^46^. Thus, even a short-term CAM assay, limited to 7–10 days incubation, offers a strong angiogenic response from a rapidly developing embryo.

The current study has a number of limitations including an inability to monitor bone cylinder vascularisation throughout the incubation process. One solution would be to introduce an *ex ovo* (shell-less) approach, although this method implies a viability drop down to 20–30% by the end of the gestational process^23^. It is worth noting that in the absence of the eggshell, the CAM can take up mineral from other sources (*i.e.* biomaterials) to restore calcium levels for skeletal development^47^. The bone resorptive potential of the CAM has been known for more than 30 years; in 1990 Webber *et al*. implanted bovine cortical bone on CAM and showed striking bone resorption following 8 days implantation^48^. In support of those findings, CAM-implanted bone cylinders resulted in a modest decrease of high densisty bone and a significant increase of low density bone, although whether this is related to any bone remodelling process, remains speculative. Future studies will include immune-detection of osteoclast markers to examine bone resorption.

## Conclusion

In summary, the present study describes a novel method to study human bone regeneration *ex vivo* using an animal model; the CAM assay. The relevance of this methodology lies in the use of human bone tissue, freshly derived from patients and hence a more clinically relevant situation to study bone healing. We suggest this human-avian platform offers an alternative animal model to test novel biomaterials and constructs for tissue engineering. This cost-effective, rapid and simple method will have a critical impact on the reduction and replacement of the number of animals used in classical *in vivo* bioengineering models. On-going studies are focusing on utilising this model for biomaterial screening, applying biomaterials in the cylindrical defect area to deliver angiogenic and osteogenic growth factors and evaluation of cell-material constructs to augment skeletal tissue formation with exciting implications for skeletal regenerative medicine.

## Materials and Methods

### Human bone cylinder extraction

**Figure 1:**
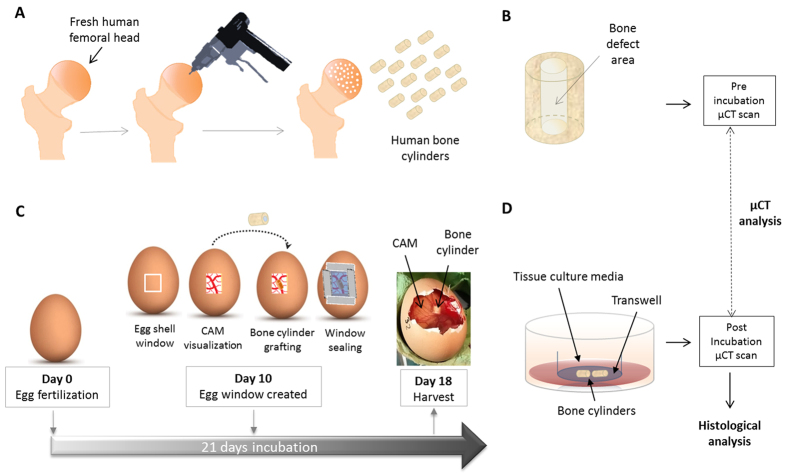
Experimental design. (**A**) Bone cylinder extraction. Human femoral heads were collected following total hip replacement surgery and bone cylinders (6 mm outer diameter, 4–6 mm length) were extracted using a surgical drill. (**B**) Engineered bone cylinders. Standard size bone cylinders were created with an empty core of 2 mm in diameter (bone defect) to facilitate CAM ingrowth and induce bone regeneration. (**C**) CAM-implantation. Fertile eggs were incubated for 10 days before removing a minimal amount of eggshell to access the CAM beneath. Bone cylinders were placed on the CAM and the eggshell was sealed to prevent infection. Incubation was resumed for an additional week before harvesting of the bone grafts. **(D**) *In vitro* incubation: organotypic culture. Bone cylinders were incubated at the air-liquid interface using a transwell membrane over one week. **Analysis**: Bone cylinders were μCT scanned before and after incubation (**C**,**D**), followed by (immuno) histochemical analysis. Chorioallantoic membrane of the chick embryo (CAM), micro computed tomography (μCT).

**Figure 2:**
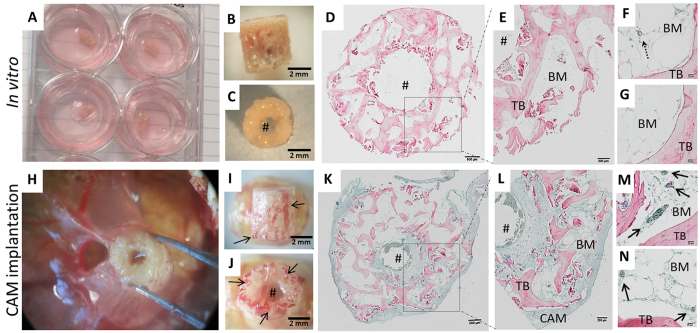
Avian blood vessels perfuse and infiltrate human bone tissue following implantation on the chorioallantoic membrane. Bone cylinders were extracted from the same femoral head immediately after surgery and incubated *in vitro* (**A**–**G**) or CAM-implanted (**G**–**N**) for 7 days. Macroscopic evaluation was performed upon harvesting of the bone cylinders (**A**–**C**,**H**–**J**), followed by histological examination (**D**–**G**, **K**–**N**). Representative paraffin-embedded cross-sections of the bone cylinders following Alcian Blue (proteoglycans) and Sirius Red staining (collagen) (**D**–**G**, **K**–**N**). Solid arrows indicate avian blood vessels. Dashed arrow indicate human blood vessel, ^#^bone defect area. Chorioallantoic membrane (CAM), bone marrow (BM), trabecular bone (TB). Scale bars detailed equivalent to 500 μm (**D**,**K**), 200 μm (**E**,**L**) and 20 μm (**E**,**G**,**M**,**N**).

**Figure 3:**
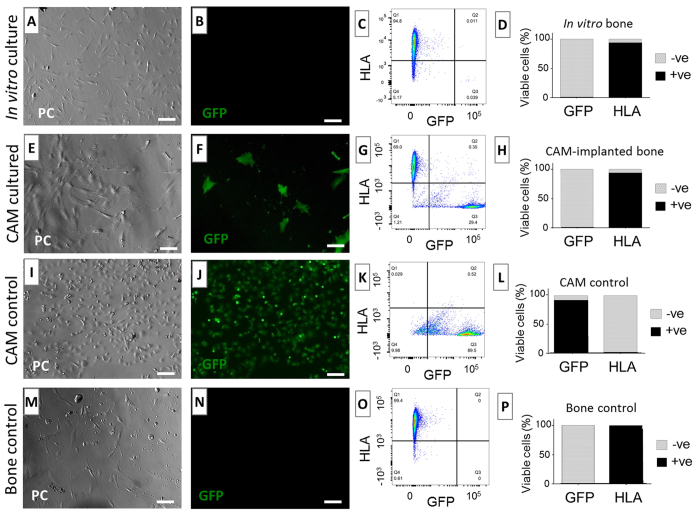
Human bone tissue remains viable following 7 days implantation on the CAM. Bone cylinders were excised and explanted in tissue culture plastic at day 0 (bone control) (**M**–**P**), day 7 of *in vitro* culture (**A**–**D**) or day 7 of GFP-CAM implantation (**E**,**F**). Data collected from two independent experiments, n = 3 cylinders per experiment. CAM only tissue was also explanted as GFP control (**I**–**L**). All conditions were imaged following 15 days culture using phase contrast (**A**,**E**,**I**,**M**) and fluorescence (**B**,**F**,**J**,**L**) to localize GFP+ cells. On confluence, cells were stained for a human specific marker (human leukocyte antigen, HLA). FACS analysis was used to interrogate for HLA+ and GFP+ cell populations (**C**,**G**,**K**,**O**). The relative number of HLA+ and GFP+ from each treatment was quantified with respect to the number of all viable cells (**D**,**H**,**L**,**P**). Phase contrast (PC), green fluorescent protein (GFP); Scale bars equivalent to 100 μm.

**Figure 4:**
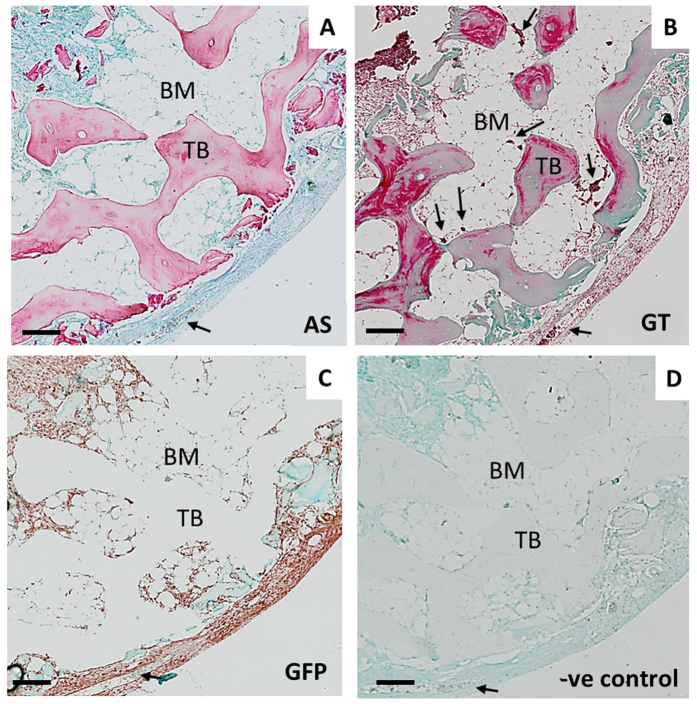
Extracellular matrix deposition localises with avian cells (GFP+) following CAM implantation. Representative images of bone cylinders implanted on the CAM for one week and then processed for paraffin histology. Consecutive sections were prepared for histochemistry; AS staining demarks proteoglycans in blue and collagen as a pink colouration (**A**), GT detects osteoid in pink, collagen in green (**B**), GFP positive immunostaining in brown-red colour, counterstained with Alcian Blue to visualize the matrix content (**C**) and isotype control (**D**). Arrows indicate blood vessels in the CAM. Human bone marrow (BM), human trabecular bone (TB), Alcian Blue and Sirius Red (AS), Goldner’s Trichrome (GT). Scale bar equivalent to 200 μm.

**Figure 5:**
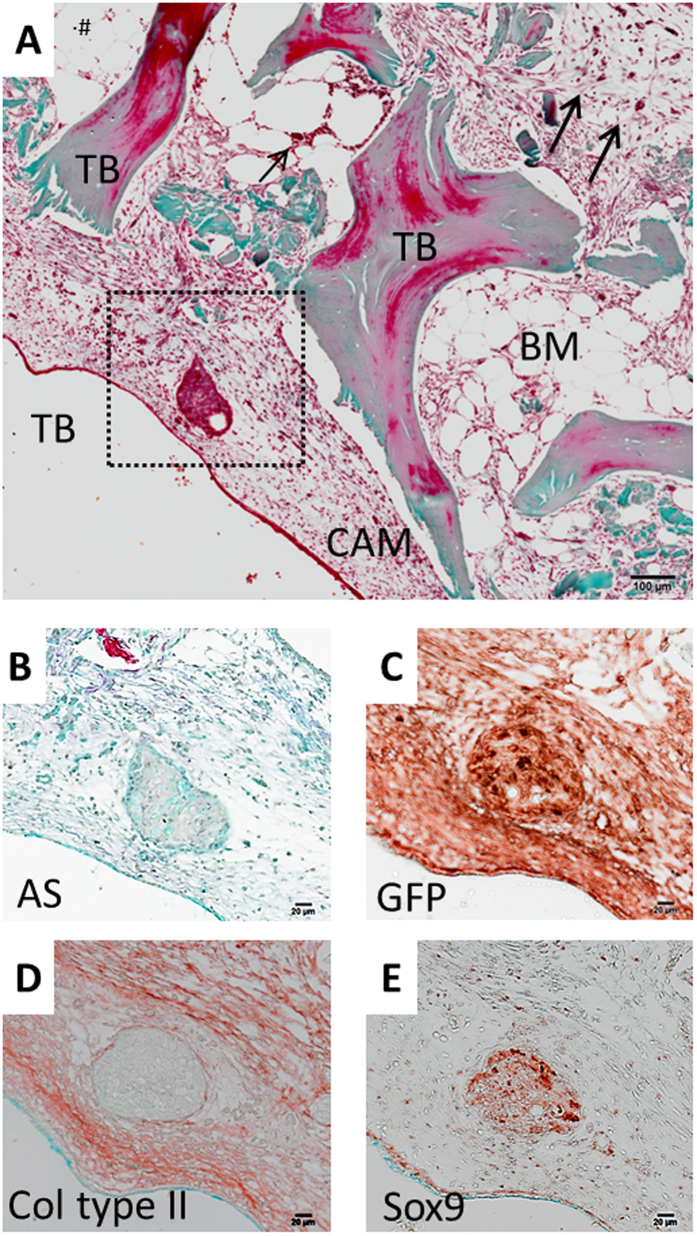
Endochondral cell condensation forms in the CAM after human bone cylinder implantation. Representative images of bone cylinders implanted on the CAM for one week and then processed for paraffin histology. Low magnification image shows the peripheral location of cell condensation in the CAM stained for GT (**A**). Cell condensation images at higher magnification stained for AS (**B**) and immunohistochemistry for GFP (**C**), Collagen type II (**D**) and Sox9 transcription factor (**E**). Arrows indicate blood vessels in the CAM. Chorioallantoic membrane (CAM), human bone marrow (BM), human trabecular bone (TB), Alcian Blue and Sirius Red (AS), Goldner’s Trichrome (GT). Representative images of CAM-implanted bone cylinders. Scale bar equivalent to 100 μm (**A**) and 20 μm (**B**–**E**).

Bone cylinders were extracted from adult femoral heads collected from haematologically normal patients undergoing routine elective hip replacement surgery. Only tissue samples that would have been discarded were used following informed consent from the patients. All protocols were conducted in accordance to the Southampton & South West Hampshire Local Research Ethics Committee (Ref: 194/99/w). All experimental protocols were approved by the Southampton & South West Hampshire Local Research Ethics Committee (Ref: 194/99/w). A dentist’s surgical drill (Osteomed, Glendale, USA) attached to a 2 mm drill bit was used to make an initial perforation. An empty-core saw (6 mm outer diameter) bound to a pilot drill (2 mm) guided the perforation of the bone cylinder outer diameter (Fig. 1A). Afterwards, forceps were used to rescue the bone cylinder (6 mm outer diameter, 2 mm empty core and 4–6 mm in length, Fig. 1B) and saline solution was perfused through its empty core to wash off any debris. A scalpel was used to remove the articular cartilage and standardize bone cylinder length. The entire protocol was conducted under aseptic conditions.

### CAM assay

All animal procedures were carried out in accordance with the guidelines and regulations laid down in the Animals (Scientific Procedures) Act 1986, UK and chick embryo chorioallantoic membrane experimental protocols were approved and conducted under Home Office Approval UK (Project licence – PPL 30/2762) approved at the University of Southampton. Assays were carried out according to fertilised green fluorescent protein (GFP) transfected eggs were kindly provided by the Transgenic Chicken Facility of the Roslin Institute^49^. The eggs were incubated horizontally for 10 days at 37 °C in a 60% humidified atmosphere, using a Hatchmaster incubator (Brinsea, UK) with one hour scheduled rotation. At day 10-post fertilisation, a fine-toothed hacksaw blade was used to make an approximate 0.5 cm^2^ square incision on the eggshell under sterile conditions and the eggshell window fragment was removed to access the CAM beneath. Bone cylinders were randomly distributed (n = 8–10 per experimental group) and individually placed on the CAM of the chicken embryo (Fig. 1C). Sterile parafilm tape was used to seal back the window and the eggs were further incubated without the rotation setting. Embryos were inspected daily by candling. After one week of incubation the bone cylinders were harvested; the CAM integration was assessed by visual inspection and photographed with a stereomicroscope attached to a digital camera (Canon Powershot G2). The number of fully developed chick embryos according to Hamburger and Hamilton^50^ was recorded and the gestational process was terminated following Home Office specific guidelines.

### Organotypic culture

Bone cylinders were placed on Millicell inserts (0.4 μm pore size, 30 mm diameter, Millipore, UK) in 6-well tissue culture plates containing serum-free basal media (αMEM, Lonza, Switzerland, with 100 U/ml 100 μg/ml of Pennicilin G and Streptomycin, PAA, USA). Bone cylinders were incubated at 37 °C and 5% CO_2_ for a week; medium was replaced every other day during that period (Fig. 1D).

### Bone viability assessment post incubation (I): cell outgrowth from explant culture

After incubation, bone cylinders and CAM tissue were collected under sterile conditions and sliced into small fragments (2–3 mm) using a scalpel. Bone fragments were evenly distributed through the surface area of a well of a 6-well-plate containing minimum amount of basal media with 10% serum to cover the surface. The plate was maintained at 5% CO_2_ at 37 °C, and the media were replaced every 5 days for the first 10 days. After 10 days, the tissue fragments were removed from the well using sterile forceps, followed by repeated 1x PBS washing steps to remove any remaining tissue. The cultures were further incubated for 10 more days with media changes every other day. Cells were trypsinised at 90% confluence and prepared for fluorescence-activated cell sorting (FACS) analysis.

### Bone viability assessment post incubation (II): Human Leukocyte Antigen (HLA) detection by FACS

Cells suspensions were washed (2x) in FACS buffer (2% foetal bovine serum and 0.1% sodium azide in PBS) before incubating with W6/32 mouse anti-human HLA-A-B-C monoclonal antibody (kindly provided by Prof. Tim Elliot, CSU, University of Southampton, UK) at 10 μg/ml, for 30 minutes at 4 °C. Following FACS buffer washes (2x), goat anti-mouse IgG (H + L)-AF647 (A-21237, Life Technologies, UK) was used for the secondary antibody incubation (1:2000 dilution) at 4 °C for 30 minutes and the cells were further washed (2x) with FACS buffer. Cells stained with secondary antibody only and non-stained cells were used as controls for non-specific antibody binding and autofluorecence, respectively; using these controls, gating for each sample was applied accordingly. A FACS Canto I Flow cytometer (BD Biosciences, San Jose, USA) was used to run the samples and data were analysed using FlowJo v10 software (Ashland, USA).

### Micro computed tomography (μCT) analysis

Bone cylinders were scanned before and after incubation using the same parameters. The bone fragments were scanned individually in sterile microcentrifuge tubes using SkyScan 1176 (Bruker, Kontich, Belgium). Bone cylinders were scanned using the following settings: X-ray source 50 kV, 500 μA, 496 ms exposure time and a voxel size of 18 μm. Once the scans were completed, the raw data was reconstructed using NRecon software with correction for misalignment, ring artefacts and beam hardening (30%). The pre- and post-implantation reconstructed datasets of the same bone graft were orientated in the same position using DataViewer software for consistent analysis. 3D registration tool of DataViewer was used to match pixels of the same density on the same location of sequential scans. Global thresholding was applied on the pre and post scans. CTAn software was employed to quantify the bone volume of each individual cylinder pre and post incubation and the relative change was calculated as a percentage of the pre scan. CTvox was implemented to create and visualise the 3D models of the bone cylinders. All the software was from SkyScan, Bruker, Kontich, Belgium.

**Figure 6:**
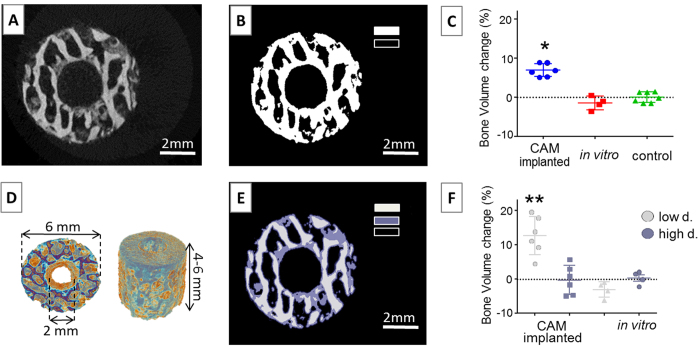
Bone volume is significantly increased following CAM implantation. Bone cylinders were scanned using the same μCT parameters before and after CAM-implantation (n = 6) or *in vitro* incubation (n = 4), or kept at 4 °C as control (n = 8). (**A**) Raw image (grayscale) of a bone cylinder μCT scan. (**B**) Standard method for image segmentation using a threshold value to differentiate mineralised tissue (white) and background (black). (**C**) Bone volume was quantified using standard binarisation method. (**D**) Three dimensional model of a bone cylinder μCT scan. (**E**) Image following the application of a three-level segmentation Otsu threshold: low dense tissue in light grey, high dense tissue in dark grey and background in black. (**F**) Bone volume was quantified following Otsu thresholding method, as previously described. Data points indicate the relative bone volume change following incubation of each individual bone cylinder. Error bars indicate mean value ± SD, *p < 0.01, **p < 0.001.

**Figure 7:**
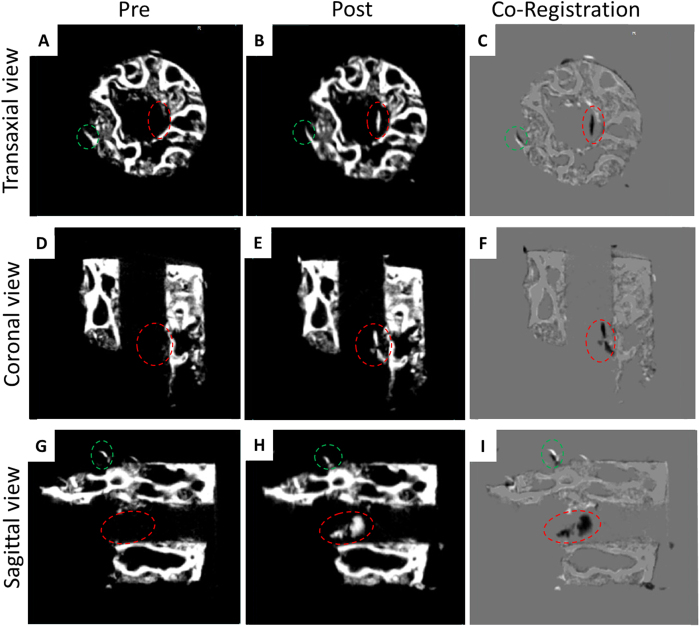
Overlay scans of the bone cylinders pre and post CAM implantation allow detection of new material deposition. Transaxial, coronal and sagittal scan images representative of bone graft before implantation on the CAM (**A**,**D**,**G**) and after (**B**,**E**,**H**), respectively. Corresponding structures in the *pre* and *post* scan are displayed in grey colour (**C**,**F**,**I**) while non-matching structures appear in white (pre) or black (post). Discontinuous circle highlight areas of interest: green indicates non-aligned structure and red indicates new deposited material.

Multi-level Otsu thresholding was applied to the scan datasets in addition to the standard analysis (global thresholding). Otsu’s algorithm consists of clustering pixels into different classes such that the variance between each class is maximal^40^. The Otsu algorithm is commonly used for image binarisation (two classes) and the same principle can be used to segment into additional classes. The present study used Otsu segmentation into two levels, separating pixels into three classes: high density, low density and background (Fig. 6E). To apply Otsu multi-level thresholding, pre and post scans of each bone cylinder were orientated on the same transaxial plane and an automated region of interest (ROI) was designed to select the full diameter. These segmented images were saved as a new dataset (Fig. 6E). Bone volume was calculated for the high density and low density classes as well as the relative change between pre and post incubation (%).

### Histology and immunohistochemistry analysis

Bone cylinders were fixed in 4% paraformaldehyde in PBS at 4 °C for 24 hours and decalcified by incubating in Histoline (Histoline, Milan, Italy) for 24 hours in rotation at 4 °C. Complete demineralisation was confirmed using flat X-rays (Faxitron). Samples were then processed for paraffin sectioning (5 μm) and stained as described by Smith *et al*.^51^ for Alcian Blue (proteoglycans) and Sirius Red (collagen) and Goldner’s Trichrome (collagen in green, osteoid in red/pink and bright red for cell cytosol). Immunohistochemistry for Sox9 and collagen type II was performed as described by Kanczler *et al*.^42^, positive staining identified in red-brown colour. GFP detection was performed using a rabbit polyclonal anti-GFP (2555, Cell Signalling, New England Biolabs, UK) at a 1:100 dilution in combination with SignalStain^®^ Boost (8114P, Cell Signalling, New England Biolabs, UK) following the manufacturer’s protocol. All immunostaining was matrix-counterstained with Alcian Blue (proteoclycans) and IgG isotype antibody was used for controls. Images were captured with an Olympus BX-51/22 dotSlide digital virtual microscope.

### Statistical analysis

All experimental data were analysed using Statistical Analysis SPSS Base 16.0 software for Windows. Results were expressed as the mean ± SD and plotted using GraphPad Prism. Normal distribution of the data was confirmed by the Kolmogorov-Smirnov test. Comparisons between treatments were performed using the one-way ANOVA test including Tukey post-hoc test. Values of p < 0.01 were considered statistically significant.

## Additional Information

**How to cite this article**: Moreno-Jimenez, I. *et al*. The chorioallantoic membrane (CAM) assay for the study of human bone regeneration: a refinement animal model in tissue engineering. *Sci. Rep.* **6**, 32168; doi: 10.1038/srep32168 (2016).

## Supplementary Material

Supplementary Information

Supplementary Video S1

## References

[b1] Cobb T. K. . Cigarette smoking and nonunion after ankle arthrodesis. Foot Ankle Int. 15, 64–67 (1994). 7981802 10.1177/107110079401500202

[b2] Macey L. R. . Defects of early fracture-healing in experimental diabetes. J. Bone Joint Surg. Am. 71, 722–733 (1989). 2659600

[b3] Robinson C. M. . Estimating the risk of nonunion following nonoperative treatment of a clavicular fracture. J Bone Jt. Surg Am 86-A, 1359–1365 (2004). 10.2106/00004623-200407000-00002 15252081

[b4] Ebraheim N. a. . Nonunion of distal femoral fractures: a systematic review. Orthop. Surg. 5, 46–50 (2013). 23420747 10.1111/os.12017 PMC6583155

[b5] Gruber R. . Fracture healing in the elderly patient. Exp. Gerontol. 41, 1080–1093 (2006). 17092679 10.1016/j.exger.2006.09.008

[b6] Hankenson K. D. . Angiogenesis in boneregeneration. Injury 42, 556–561 (2011). 10.1016/j.injury.2011.03.035 PMC3105195 21489534

[b7] Giannoudis P. V., Einhorn T. a. & Marsh D. Fracture healing: The diamond concept. Injury 38, S3–S6 (2007). 10.1016/s0020-1383(08)70003-2 18224731

[b8] Tuli R. . Characterization of multipotential mesenchymal progenitor cells derived from human trabecular bone. Stem Cells 21, 681–693 (2003). 14595128 10.1634/stemcells.21-6-681

[b9] Wall R. J. & Shani M. Are animal models as good as we think? Theriogenology 69, 2–9 (2008). 17988725 10.1016/j.theriogenology.2007.09.030

[b10] Greek R. & Menache A. Systematic reviews of animal models: Methodology versus epistemology. Int. J. Med. Sci. 10, 206–221 (2013). 23372426 10.7150/ijms.5529 PMC3558708

[b11] Perel P. . Comparison of treatment effects between animal experiments and clinical trials: systematic review. BMJ 334, 197 (2007). 17175568 10.1136/bmj.39048.407928.BE PMC1781970

[b12] Russel W. M. S. & Burch R. The Principles of Humane Experimental Technique. London, Methuen, (1959).

[b13] Nowak-Sliwinska P. . The chicken chorioallantoic membrane model in biology, medicine and bioengineering. Angiogenesis 17, 779–804 (2014). 25138280 10.1007/s10456-014-9440-7 PMC4583126

[b14] Ribatti D. . The chick embryo chorioallantoic membrane as a model for *in vivo* research on anti-angiogenesis. Curr. Pharm. Biotechnol. 1, 73–82 (1996). 11467363 10.2174/1389201003379040

[b15] Cantatore F. P. . Osteocalcin is angiogenic *in vivo*. Cell Biol. Int. 29, 583–585 (2005). 15979904 10.1016/j.cellbi.2005.03.011

[b16] Ribatti D., N. . The gelatin sponge-chorioallantoic membrane assay. Nat. Protoc. 1, 85–91 (2006). 17406216 10.1038/nprot.2006.13

[b17] Kunzi-Rapp K. . Characterization of the chick chorioallantoic membrane model as a short-term *in vivo* system for human skin. Arch. Dermatol. Res. 291, 290–295 (1999). 10367712 10.1007/s004030050410

[b18] Sys G. . Tumor grafts derived from sarcoma patients retain tumor morphology, viability, and invasion potential and indicate disease outcomes in the chick chorioallantoic membrane model. Cancer Lett. 326, 69–78 (2012). 22841668 10.1016/j.canlet.2012.07.023

[b19] Ribatti D. . Angiogenic response induced by acellular brain scaffolds grafted onto the chick embryo chorioallantoic membrane. Brain Res. 989, 9–15 (2003). 14519506 10.1016/s0006-8993(03)03225-6

[b20] Friend J. V. . Immaturity of the inflammatory response of the chick chorioallantoic membrane. Toxicol. Vitr. 4, 324–326 (1990). 10.1016/0887-2333(90)90074-4 20702188

[b21] Lafferty K. J. . Reactions of the graft versus host in the CAM. Aust. J. Exp. Biol. Med. Sci. 47, 17–54 (1969). 4890818 10.1038/icb.1969.3

[b22] Valdes T. I. . The chick chorioallantoic membrane as a novel *in vivo* model for the testing of biomaterials. J. Biomed. Mater. Res. 62, 273–282 (2002). 12209948 10.1002/jbm.10152

[b23] Valdes T. I. . Ex ova chick chorioallantoic membrane as a novel *in vivo* model for testing biosensors. J. Biomed. Mater. Res. A 67, 215–223 (2003). 14517879 10.1002/jbm.a.10055

[b24] Keshaw H. . Microporous collagen spheres produced via thermally induced phase separation for tissue regeneration. Acta Biomater. 6, 1158–1166 (2010). 19733702 10.1016/j.actbio.2009.08.044

[b25] DeVolder R. J. . Microfabrication of proangiogenic cell-Laden alginate-g-Pyrrole hydrogels. Biomaterials 33, 7718–7726 (2012). 22840222 10.1016/j.biomaterials.2012.07.001

[b26] Hadjizadeh A. . Directional migration of endothelial cells towards angiogenesis using polymer fibres in a 3D co-culture system. J. Tissue Eng. Regen. Med. 4, 524–531 (2010). 20872739 10.1002/term.269

[b27] Diaz-Gomez L. . Biodegradable electrospun nanofibers coated with platelet-rich plasma for cell adhesion and proliferation. Mater. Sci. Eng. C 40, 180–188 (2014). 10.1016/j.msec.2014.03.065 PMC4051303 24857481

[b28] Kanczler J. M. . Supercritical carbon dioxide generated vascular endothelial growth factor encapsulated poly(dl-lactic acid) scaffolds induce angiogenesis *in vitro*. Biochem. Biophys. Res. Commun. 352, 135–141 (2007). 17112464 10.1016/j.bbrc.2006.10.187

[b29] Buschmann J. . Tissue engineered bone grafts based on biomimetic nanocomposite PLGA/amorphous calcium phosphate scaffold and human adipose-derived stem cells. Injury 43, 1689–1697 (2012). 22769980 10.1016/j.injury.2012.06.004

[b30] Yang X. B. . Human osteoprogenitor bone formation using encapsulated bone morphogenetic protein 2 in porous polymer scaffolds. Tissue Eng. 10, 1037–1047 (2004). 15363161 10.1089/ten.2004.10.1037

[b31] Holzmann P. . Investigation of bone allografts representing different steps of the bone bank procedure using the CAM-model. ALTEX 27, 97–103 (2010). 20686742 10.14573/altex.2010.2.97

[b32] Kern S. . Comparative analysis of mesenchymal stem cells from bone marrow, umbilical cord blood, or adipose tissue. Stem Cells 24, 1294–1301 (2006). 16410387 10.1634/stemcells.2005-0342

[b33] Borges J. . Chorioallantoic membrane angiogenesis model for tissue engineering: a new twist on a classic model. Tissue Eng. 9, 441–450 (2003). 12857412 10.1089/107632703322066624

[b34] Martinez-Madrid B. . Chick embryo chorioallantoic membrane (CAM) model: a useful tool to study short-term transplantation of cryopreserved human ovarian tissue. Fertil. Steril. 91, 285–292 (2009). 18291379 10.1016/j.fertnstert.2007.11.026

[b35] Bellairs R. & Osmond M. *Atlas of Chick Development*. *Atlas Chick Dev.* (2014).

[b36] Akiyama H. . The transcription factor *Sox9* has essential roles in successive steps of the chondrocyte differentiation pathway and is required for expression of *Sox5* and *Sox6*. Genes Dev. 16, 2813–2828 (2002). 12414734 10.1101/gad.1017802 PMC187468

[b37] Hancox N. M. The survival of transplanted embryo bone grafted to CAM and subsequent osteogenesis. J. Physiol. 106, 279–285 (1946). 16991760 10.1113/jphysiol.1947.sp004211 PMC1393790

[b38] Oryan A. . Bone regenerative medicine: classic options, novel strategies, and future directions. J. Orthop. Surg. Res. 9, 18 (2014). 24628910 10.1186/1749-799X-9-18 PMC3995444

[b39] Hopkins D. R. . The bone morphogenetic protein 1/Tolloid-like metalloproteinases. Matrix Biol. 26, 508–523 (2007). 17560775 10.1016/j.matbio.2007.05.004 PMC2722432

[b40] Otsu N. . A Tlreshold Selection Method from Gray-Level Histograms. IEEE Trans. Syst. man Cybern. 20, 62–66 (1979).

[b41] Diyana W. M. . Multi-level segmentation method for serial computed tomography brain images. Signal Image Process. Appl. (ICSIPA), 2009 IEEE Int. Conf. 107–112 (2009).

[b42] Kanczler J. M. . A novel approach for studying the temporal modulation of embryonic skeletal development using organotypic bone cultures and microcomputed tomography. Tissue Eng. Part C. Methods 18, 747–760 (2012). 22472170 10.1089/ten.tec.2012.0033 PMC3460619

[b43] Shah N. J. . Adaptive growth factor delivery from a polyelectrolyte coating promotes synergistic bone tissue repair and reconstruction. Proc. Natl. Acad. Sci. USA 111, 1–6 (2014). 10.1073/pnas.1408035111 PMC4156697 25136093

[b44] Yuasa M. . The temporal and spatial development of vascularity in a healing displaced fracture. Bone 67, 208–221 (2014). 25016962 10.1016/j.bone.2014.07.002

[b45] Thompson T. J. . Intramembranous osteogenesis and angiogenesis in the chick embryo. J. Anat. 166, 55–65 (1989). 2482839 PMC1256739

[b46] Steffens L. . *In vivo* engineering of a human vasculature for bone tissue engineering applications. J. Cell. Mol. Med. 13, 3380–3386 (2009). 18624770 10.1111/j.1582-4934.2008.00418.x PMC4516493

[b47] Vargas G. E. . Biocompatibility and bone mineralization potential of 45S5 Bioglass^®^-derived glass-ceramic scaffolds in chick embryos. Acta Biomater. 5, 374–380 (2009). 18706880 10.1016/j.actbio.2008.07.016

[b48] Webber D. M. . An *in vivo* model system for the study of avian osteoclast recruitment and activity. Bone Miner. 11, 127–140 (1990). 2268742 10.1016/0169-6009(90)90053-i

[b49] Simon G. . Transgenic chickens as bioreactors for protein based drugs. Drug Discov. Today 3, 1–9 (2005). 10.1016/S1359-6446(04)03317-3 15708533

[b50] V. Hamburger H. L. H. A series of normal stages in the development of the chick embryo. J. Morphol. 88, 49–92 (1951). 24539719

[b51] Smith E. L. . Evaluation of skeletal tissue repair, Part 1: Assessment of novel growth-factor-releasing hydrogels in an *ex vivo* chick femur defect model. Acta Biomater. 10, 4186–4196 (2014). 24937137 10.1016/j.actbio.2014.06.011

