# Supplementary material for: The chorioallantoic membrane (CAM) assay for the study of human bone regeneration: a refinement animal model for tissue engineering
Source: Sci Rep. 2016 Aug 31;6:32168. doi: 10.1038/srep32168 (PMC5006015; doi:10.1038/srep32168)
Supplement: Supplementary Information [file srep32168-s1.pdf]

Supplementary Information

Title: The chorioallantoic membrane (CAM) assay for the study of human bone regeneration: a refinement animal model in tissue engineering.

Authors: Ines Moreno-Jimenez[1], Gry Hulsart-Billstrom[1], Stuart A. Lanham[1], Agnieszka A. Janeczek[1], Nasia Kontouli[2], Janos M. Kanczler[1], Nicholas D. Evans[1] and Richard OC Oreffo*[1].

Bone and Joint Research Group, [1]Centre for Human Development, Stem Cells and Regeneration, Human Development and Health, Institute of Developmental Sciences University of Southampton, Tremona Road, Southampton, SO16 6YD, UK

[2]Cancer Sciences Unit, Institute for Developmental Sciences, University of Southampton, Tremona Road, Southampton, SO16 6YD, UK

Supplementary Table S1: Chick embryo viability following 7 day CAM implantation of live human bone in four independent experiments (Exp 1 to 4). Each experiment conducted on an individual femoral head from a patient undergoing total hip replacement surgery.

	**Viable embryos**	**Total number of embryos**	**Percentage of viable embryos**
Exp 1	10	10	100%
Exp 2	7	10	70%
Exp 3	4	8	50%
Exp 4	7	10	70%
		**Mean**	**72.5%**
		**SD**	**20.6**

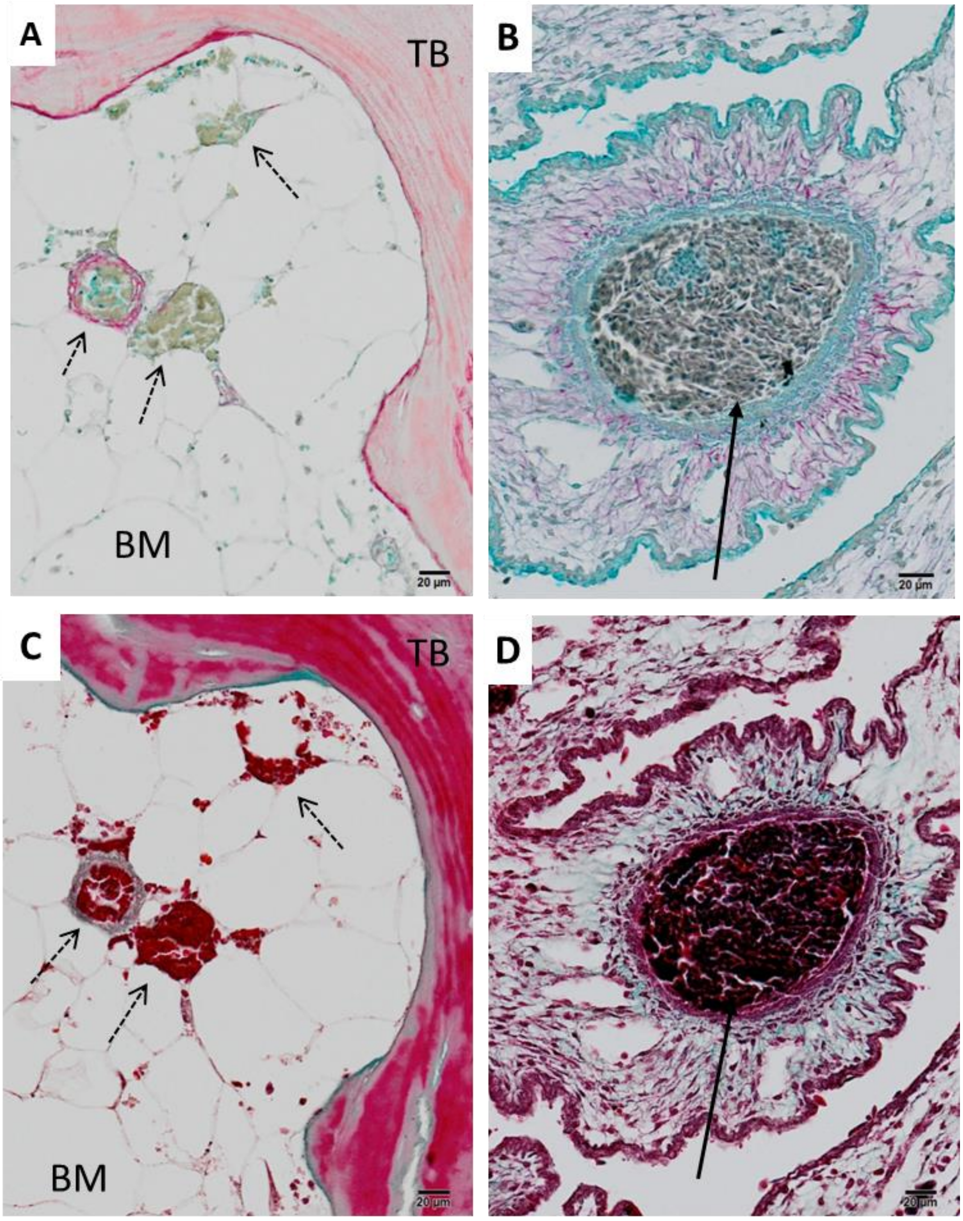

Supplementary Figure S1: Histological differences between human and avian erythrocytes. Paraffin sections of freshly isolated human bone (A and C) and CAM tissue (B and D) stained for Alcian Blue and Sirius Red (A-B) and parallel section for Goldner's Trichrome (C-D). Dashed arrows indicate round and enucleated human erythrocytes in the bone marrow. Solid arrows indicate chick erythrocytes in a CAM blood vessel. Scale bars detailed for each picture.

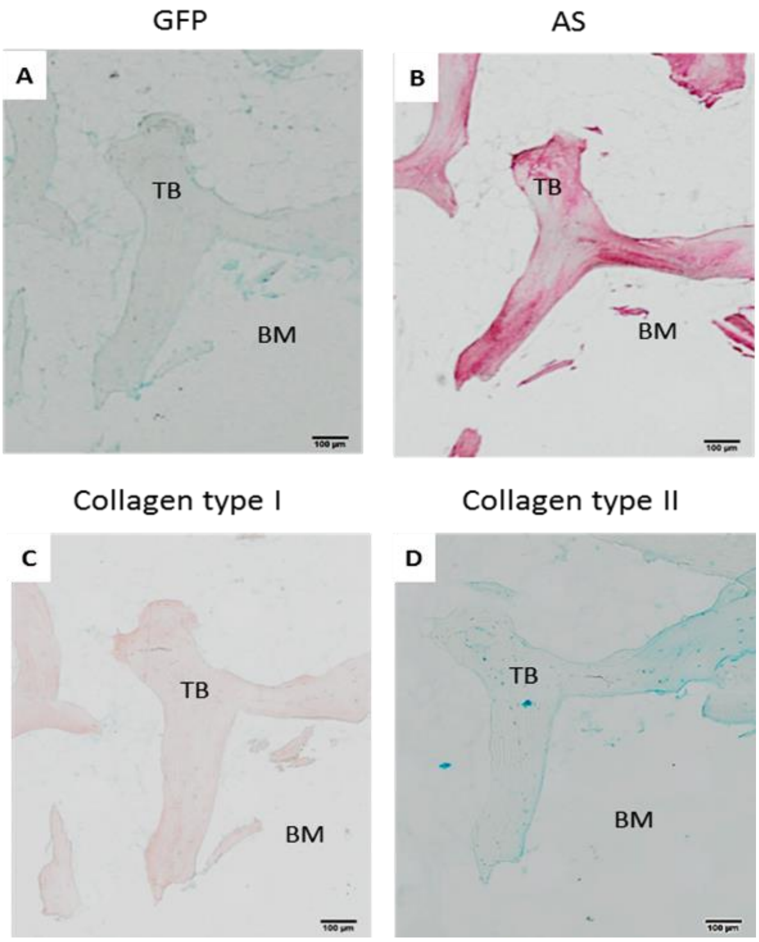

Supplementary Figure S2: Absence of collagen deposition in bone cylinders cultured *in vitro* for 7 days. Bone cylinders were cultured *in vitro* for one week and then processed for paraffin histology. Parallel sections were stained for Alcian Blue and Sirius Red (B), and immunohistochemistry for GFP (A), Collagen type I (C) and Collagen type II detection (D). Immunohistochemistry was counterstained using Alcian Blue to visualise the matrix content. Human bone marrow (BM), human trabecular bone (TB), Alcian Blue and Sirius Red (AS). Scale bars detailed in each picture.

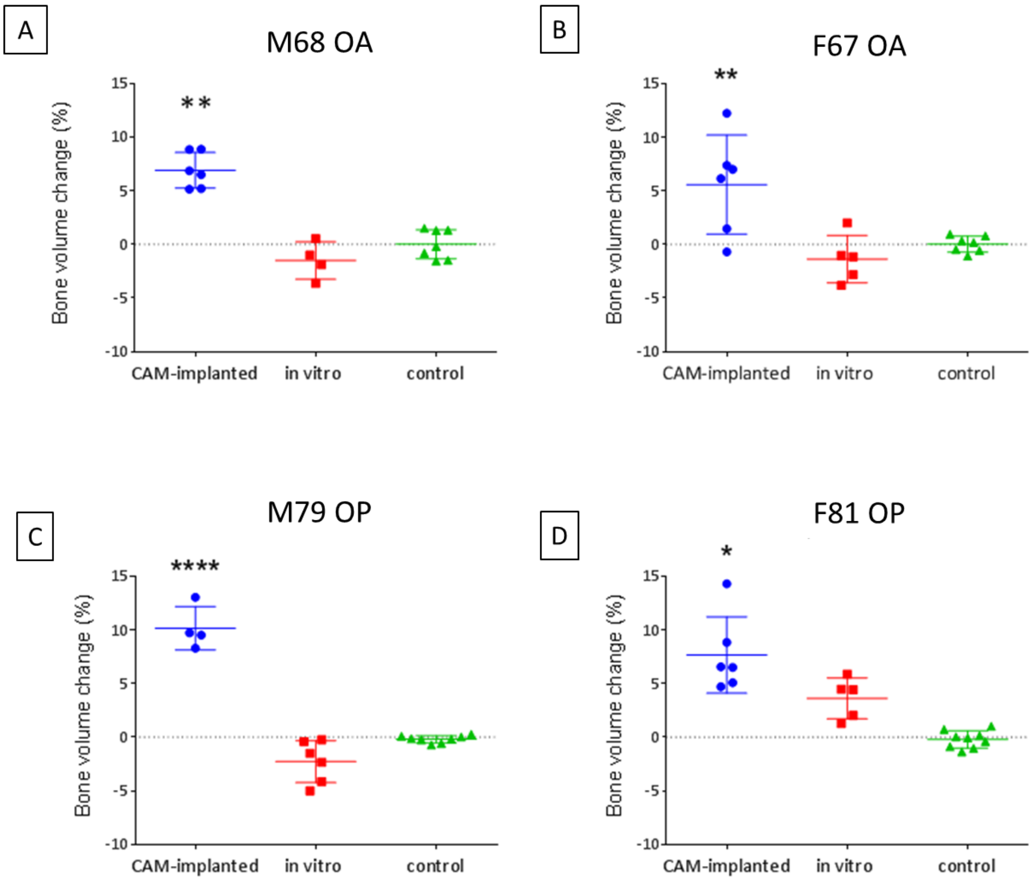

Supplementary Figure S3: Consistent significant bone volume increase following CAM-implantation. Bone cylinders were scanned using the same µCT parameters before and after CAM-implantation (n=4-8) or *in vitro* incubation (n=4-6), or kept at 4˚C as control (n=8). Femoral heads from osteoarthritic (OA) and osteoporotic (OP) patients were used in four independent experiments: (A) Male 68 years old OA, (B) Female 67 years old OA, (C) Male 76 years old OP and (D) Female 81 years old OP. Bone volume was quantified using standard binarisation method. Data points indicate the relative bone volume change following incubation of each individual bone cylinder. Error bars indicate mean value ± SD, *p<0.01, **p<0.001, ** **p<0.00001.

Supplementary Video: Incorporation of the human bone cylinder on CAM following 7 days implantation. The bone cylinder integrated with avian blood vessels, as shown by the beating micro-movement of the CAM circulatory system at the moment of harvest (chick embryo day 18).
